# Childhood cancer research in Oxford I: the Oxford Survey of Childhood Cancers

**DOI:** 10.1038/s41416-018-0180-0

**Published:** 2018-08-21

**Authors:** JF Bithell, GJ Draper, T Sorahan, CA Stiller

**Affiliations:** 1 Department of Statistics, University of Oxford, 24-29 St Giles’, Oxford, OX1 3LB UK; 2 Institute of Applied Health Research, College of Medical and Dental Sciences, University of Birmingham, Edgbaston, Birmingham B15 2TT UK; 3 National Cancer Registration and Analysis Service, Public Health England, 4150 Chancellor Court, Oxford, OX4 2GX UK

**Keywords:** Risk factors, Oncology

## Abstract

**Background:**

Significant research on the epidemiology and natural history of childhood cancer took place in the Universities of Oxford and Birmingham over sixty years. This is the first of three papers recording this work and describes the Oxford Survey of Childhood Cancers (OSCC), the largest case-control survey of childhood cancer ever undertaken.

**Methods:**

The OSCC studied deaths in Britain from 1953 to 1981. Parents were interviewed and medical records from ante-natal clinics and treatment centres were followed up and abstracted. The survey left Oxford in 1975 and was run subsequently from Birmingham. The data are now being documented and archived to make them available for future study.

**Results:**

Many papers have resulted from this survey, most notably those relating to the association first reported therein between childhood cancer and ante-natal X-raying. This paper is a historical review of the OSCC.

**Conclusions:**

In spite of many analyses of the study, this historic data set has continuing value because of the large number of examples of some very rare tumours and the detailed clinical and family history data that are available; and also because of the possibility of carrying out new analyses to investigate emerging research issues.

## Introduction

This paper is the first of three describing the work done on childhood cancer in Oxford over six decades between 1954 and 2014. The intention of these papers is to summarise the history and achievements and to record the current availability of the very substantial research resources accumulated over this period. This first paper describes the genesis and achievements of the first part of the work, the Oxford Survey of Childhood Cancers (OSCC). The second of these papers^1^ describes the extension of the work of the OSCC by the Childhood Cancer Research Group (CCRG), though the work on ionising radiation is dealt with in a separate, third paper.^2^

The OSCC was started by a remarkable woman, Dr Alice Stewart. She was born in Sheffield in 1906, the daughter of two progressive liberal doctors, from whom she inherited a life-long passion for social justice and an almost iconoclastic attitude to established beliefs. She studied medicine at Cambridge, an uncomfortable place for a female medical student in 1920s, and completed her training at the Royal Free Hospital, where she established herself as a brilliant young diagnostician. She came to Oxford in 1941, initially working under Dr Leslie Witts, but was soon appointed to the new Institute of Social Medicine. This was set up under Professor John Ryle, who had given up a prestigious chair in Cambridge to work in the new discipline—a large part of which was concerned with what we would now call epidemiology. When Ryle died in 1950, Stewart had started to work on his Child Health Study and in particular had decided to investigate the causes of childhood leukaemia; at that time this disease was perceived to be increasing in incidence; this could well have been partly because antibiotics were curing infectious diseases such as pneumonia that would previously have masked an underlying tumour.^3^

Realising that the disease was so rare that following a cohort of children would require a prohibitively large study to detect associations, Stewart embarked instead on a case-control study, itself so ambitious as to deter most scientists, for which she obtained the death certificates of all children dying of the disease in England or Wales. Each was matched with a healthy control child and—after an interval of two (later three) years—the respective mothers were interviewed by medical staff recruited from local authorities.

By any standards, the survey involved an impressive degree of organisation and would be extremely difficult to repeat in modern times owing to data protection and other legal considerations. Initially, only children dying of malignant disease before age 10 were included, though this was later extended to ages up to 16 and to include Scotland. Each control child was the first available on a ‘control selection list’ of children matched by sex and date of birth that was compiled from the birth register for the area in which the index child—or ‘case’—had died. This enabled the same interviewer to see the parents of both children for the majority of case–control pairs. The first interviews were for children dying in 1953 and their controls. The first major publication^4^ analysed over 1400 case–control pairs for children dying in the years 1953 to 1955. The principal finding was an association between cancer or leukaemia and irradiation of the foetus in an ante-natal X-ray.

This paper describes the development of the survey as it moved from Oxford to Birmingham, its relationship to the CCRG, the scope and limitations of the data collected and some notable publications describing its principal findings. A discussion considers its significance for our understanding of childhood cancer and its scope for further insights. A biography of Alice Stewart was published shortly before she died in 2002;^5^ this should be read in conjunction with a scientific appraisal by Wakeford.^6^

## Materials and methods

### Development of the survey

The association with foetal exposure to X-rays was controversial and inevitably ensured continuing work on the survey. In 1962 there was a new development, in that national cancer registration became fully functional throughout Great Britain; for England and Wales see Swerdlow;^7^ for Scotland see Boyle, Robertson.^8^ From that point onwards the Oxford survey team started to collect registration information for children who had survived a cancer other than leukaemia for at least three years, forming the so-called ‘Live Series’. It was, however, impossible to find satisfactory controls for the surviving children, and the original study design—based on ascertainment at death—was continued.

In 1969 Professor Richard Doll was appointed to the Regius Chair of Medicine in Oxford. Unfortunately, he and Stewart had disagreed publicly and vehemently about the association between childhood cancer and foetal X-raying—mostly on the grounds that the survey could not rely on accurate and equal recollection of hospital episodes by case and control mothers. In fact, the greatest care had been taken to minimise the potential for case-control bias, notably by checking the mothers’ claims against hospital or clinic records. Nevertheless widespread scepticism remained, partly driven no doubt by reluctance to accept any danger attached to a widespread and valuable diagnostic tool, but also because a cohort study^9^ had failed to confirm the association. The disagreement between Doll and Stewart—exacerbated by the latter’s pugnacious defence of her findings—meant that, when Stewart reached retirement age in 1974, it was virtually inevitable that she would be unable to continue her work in Oxford. She therefore accepted a research fellowship in the Department of Social Medicine at Birmingham University and the original survey data left Oxford, initially to the Marie Curie Foundation in Limpsfield, who had kindly agreed to host the data collection. Later the operation moved to the Department of Social Medicine in Birmingham; her colleagues Margaret Kinnier Wilson and George Kneale also left Oxford to work in Birmingham. Stewart and her colleagues continued to publish analyses of the survey for some while after data collection ceased, with deaths for the year 1981, though she increasingly turned her attention to other investigations concerned with ionising radiation. The data were later looked after in the School of Health and Population Sciences at Birmingham University by George Knox and Tom Sorahan and papers continued to appear for twenty years. These included further analyses of the ante-natal X-raying data—a subject that remained controversial, though Doll came to accept that the association was probably causal,^10^ not least because of doubts about the cohort study, which in any case had limited power. A good account of the controversy over the causal nature of the association is given by Wakeford.^11^

**Fig. 1:**
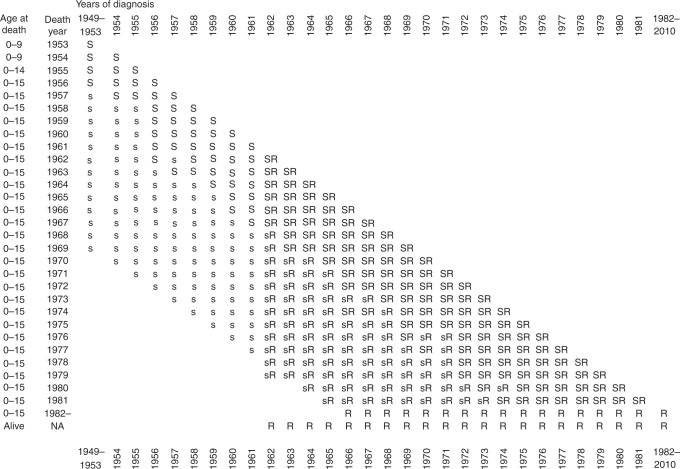
Relationship between the NRCT and the OSCC. R denotes year combinations in which cases were registered in the NRCT; S,s indicate those in which cases could be ascertained in the OSCC, the latter (s) indicating years in which there were fewer than 5 cases observed. The predominance of years marked s results from improving treatment and short-term survival

With Stewart’s departure to Birmingham, the staff and computing resources in Oxford were redeployed to form the CCRG, with the support of the Department of Health, as described in Draper, Bithell, Bunch, Kendall, Murphy, Stiller.^1^ The data were reorganised to form the National Registry of Childhood Tumours (NRCT), with ascertainment by registration and so including an increasing proportion of survivors; the earlier dead cases from the OSCC were included in the register, while new ones were notified by the CCRG to the OSCC. Figure 1 shows in schematic form the relationship between the NRCT and the OSCC by tabulating the years of diagnosis and death in which data are available.

### Scope and limitations of the OSCC data

**Fig. 2:**
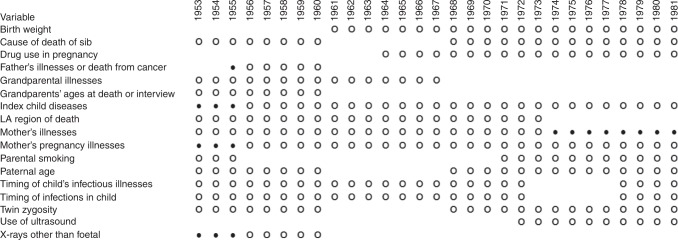
Coverage of some of the more important variables in the OSCC; **O** indicates virtually complete ascertainment by design, i.e., complete apart from cases that could not be traced for some reason; • indicates partial ascertainment

The main dataset is currently being checked and documented with a view to archiving it, after which it is hoped to make it generally available, subject to the restrictions entailed by data protection legislation and ethical compliance. It contains records of 23,764 cases and their controls, though only 14,938 (63%) of the cases were adequately traced and interviewed. The original survey data were abstracted from interview forms, copied into ledgers—a system designed before the availability of electronic computers—and only transferred to early computers from the late 1960s. Provision was made in the data record for over 200 variables, though many of the fields were largely empty since they were concerned with recording many possibilities that were not necessarily applicable, for example the disease experience of the children’s relatives. Furthermore, not all the fields were abstracted throughout the study period: typically, questions would be dropped from successive versions of the interview schedule when analyses suggested that they were unimportant, while new questions would be added to pursue new investigations. Figure 2 shows the coverage by years of some of the more important variables in the OSCC, distinguishing years of death in which there was virtually complete and only partial ascertainment.

### Information coded is available on various topics, including

#### Birth details

Sex and sex of co-twin if a twin, position in the sibship and any significant congenital abnormalities. Birth weight is recorded only from 1961. Data for later years were used in two of the papers on parental smoking discussed below; no differences in mean birth weights between cases and controls were observed.

#### Diagnosis

Cases were originally coded to a four-point pathology code based on the International Classification of Diseases, Sixth edition,^12^ for the years 1953–73. This coding was supplemented by a 4-point code using information from medical records and indicating tumour site (in terms of anatomical system), tissue type and tumour position. For leukaemia, an alternative four-point code was used, giving information on the leucocyte count, the predominant cell type, the percentage of cells of the predominant type, and the predominant type ascertained from any marrow biopsy; of these, just the leucocyte count was preserved throughout the study. After 1973 the ICD coding was replaced by MOTNAC,^13^ a system recording tumour type and site. All cases have now all been coded also to the groups and subgroups of the International Classification of Childhood Cancer, third edition (ICCC3),^14^ though for deaths before 1962 the most detailed level, the “divisions”, are not available. ICCC3 is based on ICD-O and is better suited to the very different pathology of tumours in children; it includes separate categories for the principal tumours believed to be of embryonic origin. For the most part, the diagnoses are taken from hospital records, though where these were unavailable or inaccessible the death certificate diagnosis was used.

#### Child’s health

Information includes details of immunisations, infections and other illnesses prior to the recorded onset of the tumour.

#### Key dates

The month and year of death are known reliably; for some cases the month of birth had to be estimated from age information, mainly for untraced cases since date of birth did not appear on death certificates before 1970. Month of onset of the tumour has been recorded throughout, though this is difficult to define clearly.

#### Local authority region at death

This was coded from a list of 244 administrative areas prevailing at the start of the study, recorded until 1973, together with a description of its urban/rural status. Some coding of birth and death addresses was carried out for geographical studies, but this has not been preserved.

#### Pregnancy X-rays

Most of the useful information on ante-natal X-raying focuses on the first of any abdominal X-ray investigations and includes the reason for the investigation, the month in the pregnancy, the number of films believed to have been exposed and details of the facility where the investigation was carried out. The mother’s account of the investigation was checked by following up the radiology records from the clinic concerned.

#### Mother’s health

This included the mother’s age and pregnancy history, her illnesses in childhood and in adult life, both before and during the relevant pregnancy; it includes drugs taken during pregnancy for deaths in 1964–79. For the years 1953–55 and 1971–1981, smoking histories of both parents were also available.

#### Family health

The age of the father and information on his illnesses and those of the sibs are available for some years, while congenital abnormalities, deaths and neoplasms in sibs were recorded for all years.

#### Socio-economic status

This was coded from the father’s occupation as recorded on the death certificate using the Registrar General’s Classification of Occupations.^15^ As this information is clearly not available for the controls, a separate coding based on the interview schedules was also recorded.

At first sight, the survey has very considerable scope for throwing light on the possible causes of childhood cancer and leukaemia, but there are distinct limitations to the data available. For one thing, the possibility of case-control recall bias means that, for many of the variables, simple case-control comparisons may not be trustworthy, though in the case of ante-natal X-raying considerable care was taken to verify the information. There is still the possibility for comparison with external data and for internal comparisons amongst the cases, looking, for example, for associations specific to particular tumours; the data do however need to be treated with considerable care. It must be remembered too that the survey was conducted over many years and inevitably the main energy of the investigators had to be expended on exploring new findings rather than checking past data and maintaining consistency of coding over successive years. Many of the interviewers and coders, though highly motivated and devoted to the aims of the survey, had not been trained in data management, with consequential scope for errors in data recording. It is also the case that the amount of information declined in the second half of the period: the number of deaths ascertained per year declined from over 1000 to around 600 between 1968 and 1981, partly because of improving survival.

Nevertheless, we feel that there is considerable useful information in the survey, not least because childhood cancer is a disease with many variants and facets and the possibility of examining small subsets in detail is of continuing value. Some of the diseases in the spectrum are very rare and the OSCC is by far the largest survey of affected children ever conducted. Unfortunately, the possibility of checking the source documents is very limited: many of the specialist forms, such as those sent to ante-natal clinics, no longer exist, though the interview forms themselves were micro-filmed and the images have since been digitised. For the cases dying in 1961-1981, hospital records still exist on paper and it is planned to scan these and incorporate them into the archive; this information is of variable quality and extent, but the records are potentially valuable in following up particular cases of interest.

## Results

### Some notable results from the survey

Since the survey began, well over a hundred contributions to the scientific literature have been made that report results from the OSCC; a list of the most important and accessible of these can be found online in the Supplementary Information. The largest number of them have related to the association with foetal exposure to ionising radiation from ante-natal X-raying.

#### Ante-natal X-raying

This association was the most significant finding in the first analyses of the survey data and it has remained so in spite of numerous investigations of other topics. The association was first reported in a Preliminary Communication in the *Lancet,*^16^ in which a statistically significant case-control excess was found amongst the first 547 case-control pairs analysed. This interim result was confirmed by Stewart, Webb, Hewitt,^4^ who presented a careful and comprehensive analysis of the 1416 traced, matched and interviewed cases dying in 1953-55. After certain other exclusions, 1299 pairs were analysed in regard to their X-ray history. For these, the case-control ratio for abdominal X-raying in the relevant pregnancy was 178/93, resulting in an estimated odds ratio of 2.06 in an unmatched analysis; the paper does not report the data in a form permitting a matched pairs analysis. Even in a careful reanalysis adjusting for possible sources of bias, the association was statistically significant (P < 0.002). The excess risk appeared to apply to malignant disease in general and already there was evidence of a systematic increase in risk with the number of films reported or estimated to have been exposed. Later estimates generally showed a decline in odds ratio over time, for example to 1.47 estimated from an analysis of 8513 pairs;^17^ these cases include older children dying under age 15 up to 1967 and the paper reported significant increases in risk for tumours other than leukaemia.

**Fig. 3:**
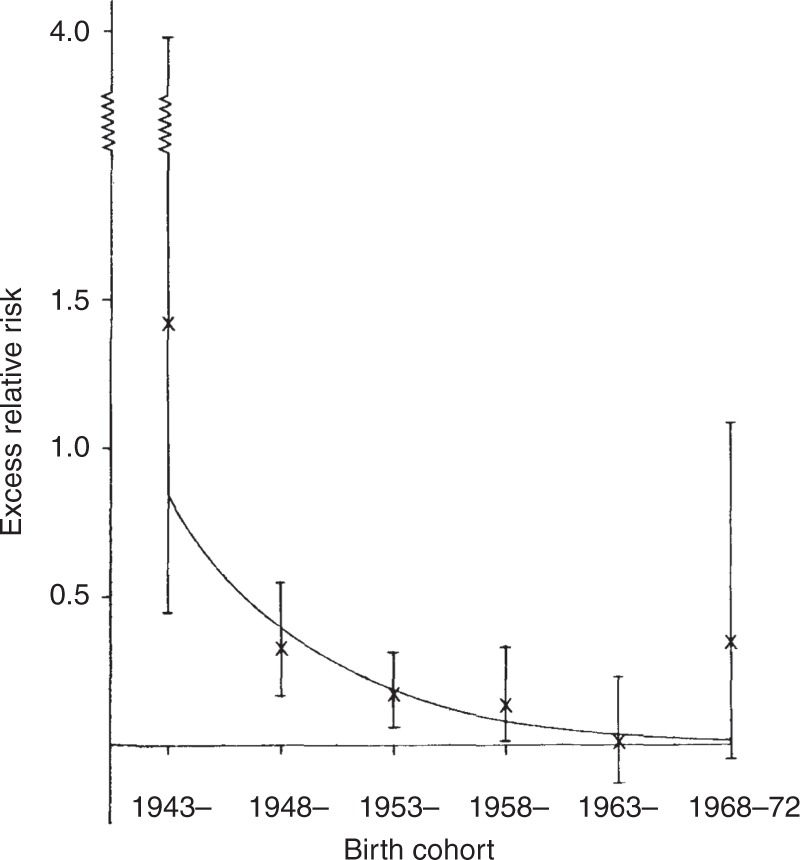
Excess relative risk per film exposed, by birth cohort, with 95% confidence limits, estimated from a multivariable model. 8513 pairs with deaths 1953–72. © 1988 by John Wiley & Son Ltd, reproduced from Bithell and Stiller^18^ by kind permission

This decline in risk is almost certainly due mainly to the lower doses delivered by the X-ray equipment in use, as is strongly suggested by Fig. 3, reproduced from Bithell, Stiller,^18^ which shows a decreasing risk per X-ray film exposed, analysed by birth cohort for deaths to 1972. The widths of the confidence intervals reflect the changing amounts of information in the different cohorts, the largest numbers of cases being in the middle of the time range; the decline is evidence supportive of a causal inference drawn from the association.

Some of the early papers reported analyses of X-ray risk using methods that were innovative though controversial; latterly, however, Kneale and his colleagues mainly used conditional logistic regression,^19^ now generally regarded as appropriate for matched case-control designs. Knox et al.^20^ used this methodology in a wide-ranging analysis of the study variables, and showed some frequency data, but report an exaggerated relative risk (RR) of 1.94 which resulted from an error in the analysis, corrected by Muirhead and Kneale^21^ and discussed by Wakeford and Little.^22^ Gilman et al.^23^ also present frequency data.

Attempts to estimate the risk per unit of radiation are frustrated by a lack of information on the radiation doses delivered by the equipment, which almost certainly varied considerably. Such information as was available at the time is comprehensively reviewed by Mole:^24^ there was clearly very considerable variation between hospitals in dose delivered, even after allowing for substantial differences over the dates of the examination and the type of procedure. A careful analysis is provided by Wakeford, Little,^22^ who estimate, albeit with very considerable uncertainty, that intrauterine exposure to X-rays caused an increase in absolute risk of cancer or leukaemia under 15 years of the order of 0.008% mGy^-1^, while Doll, Wakeford^10^ assess the evidence in the light of controversial issues raised. Gilman, Stewart, Knox, Kneale^25^ give an overview of the changes in obstetric practice over the period of the study and demonstrate the increasing use of ultrasound investigations from 1972, for which Kinnier Wilson, Waterhouse^26^ found no evidence of an associated carcinogenic risk.

The controversy referred to above, which led to delayed acceptance of the causative nature of the association observed, resulted in part from criticisms of the case-control design of the survey, though these were largely allayed by the paper of MacMahon,^27^ who found similar results to the OSCC in a hospital-based survey with a design that avoided recall bias. There was also an issue of compatibility of the OSCC estimates with those of other studies, notably estimates obtained by extrapolating from higher doses in the studies of the survivors of the atomic bombing of Japan. Most of the latter information relates to post-natal exposure, which may not entail the same risk as for embryonic exposure. Recent studies of children exposed to CT scans have also provided some evidence of risk to juvenile tissue from low dose radiation in a range comparable with the OSCC findings.^28^ Wakeford^29^ discusses the compatibility of these leukaemia risk estimates; it is becoming clear that the OSCC finding of radiation risk at low doses can no longer be dismissed as an isolated observation resulting from a flawed methodology.

#### First comprehensive analysis

The first major publication,^4^ referred to above, analysed just the 1416 cases dying in England and Wales from 1953-55 under 10 years of age, the survey age-range being extended subsequently. The paper gave a model analysis of the data, with hand calculations that precluded the more sophisticated statistical methodology now available, but nevertheless examined possible biases and confounding factors using ingenious comparisons that are still well worth studying. For example, where a subgroup showed an excess of cases over controls, the authors checked to see if the individuals involved were also more likely to show a difference in reporting information unlikely to be related to cancer; they generally found consistency between cases and controls. In addition to their analysis of ante-natal X-raying, described above, they drew attention to many of the associations that were the subject of subsequent papers involving more cases and demonstrated early indications of significant associations. Thus, for example, they found reports of serious maternal virus infections in 10 cases (of rubella, mumps, herpes zoster or infective hepatitis) but only one control record; the numbers were too small for individual disease comparisons to achieve statistical significance. Importantly, they also highlighted the absence of a case-control difference in maternal health before the relevant pregnancy, which argues against the possibility that childhood cancer might be largely determined by an inherited tendency to morbidity or lowered immunocompetence. Other analyses in the paper concern the child’s other illnesses, treatment and any congenital abnormalities; the family history, including the occurrence of neoplasms in close relatives; and post-natal X-ray exposure of the child. There was no case-control excess for post-natal diagnostic X-rays; the numbers of cases (8) and controls (3) treated with therapeutic X-ray treatments were too small to draw useful conclusions.

#### Progress reports

A series of progress reports were published in successive years from 1963 to 1966 in the *Monthly Bulletin of the Ministry of Health and the Public Health Laboratory Service*. These dealt with various particular topics, including the completeness of ascertainment of birth cohorts,^30^ the occurrence of congenital abnormalities and deaths in sibs,^31^ the association of childhood leukaemia and Down syndrome,^32^ and the comparative reliability of case and control reporting.^33^ These reports make interesting reading, though they cover deaths only to 1960 and have to some extent been superseded by later publications. They are unfortunately not currently available in digital form on the web.

#### The role of infectious organisms

Following a report of a considerable excess of mothers in the National Child Development Study (NCDS) cohort who were exposed to influenza in pregnancy and whose children developed leukaemia,^34^ Bithell et al.^35^ carried out an analysis of maternal virus infections in the OSCC for 9074 children dying in 1953–67. Of the associations with maternal virus infections during pregnancy reported by Stewart, Webb, Hewitt^4^ and referred to above, only rubella showed a case-control excess, with 17 cases to 7 controls. Significant excesses for chicken pox and influenza were also observed, though the estimated odds ratio for the latter of 1.52 (95% confidence limits 1.11, 2.14) was appreciably less than that observed in the NCDS cohort, whose mothers were exposed to a particularly virulent ‘Asian’ strain of the virus in the winter of 1957–58. In an examination of later OSCC data, Blot et al.^36^ found no association with chicken pox but did report a persistent case-control excess of maternal rubella infection.

#### Mother’s illnesses and drugs taken in pregnancy

The data show appreciable excesses of reported illnesses and drugs administered among the cases compared with the controls,^37^ but interpreting these is particularly difficult because of the possibility of recall bias and also the problem of distinguishing the effects of the illness and the treatment. Thus Sanders, Draper^38^ examined the prevalence of pulmonary tuberculosis and epilepsy, both appreciably more frequent in case mothers than in controls. They demonstrated, however, that the proportions of mothers affected by illness who were prescribed certain drugs, in particular isoniazid and phenytoin, were similar between cases and controls, suggesting that association could be attributed to the disease rather than the treatment. In a more comprehensive study, Gilman et al.^39^ presented an analysis of all recorded drugs and illnesses using logistic regression, which effectively adjusted estimates of individual drug or illness effects for overall case-control reporting differences. They concluded that the effects of drugs taken during pregnancy were secondary to those of certain illnesses, notably viral infections and other illnesses involving pyrexia. The only drug groups with consistent residual effects in the analysis were analgesics, antipyretics and vaccines.

#### Parental tobacco and alcohol consumption

A study of 1641 matched pairs for the years 1977–81^40^ revealed no important effect of parental alcohol consumption or maternal smoking on childhood cancer risk, but a highly significant trend with tobacco use by the children’s fathers (*P* < 0.001), confirming an association found from other, smaller studies. This trend was also confirmed in analyses of data from the OSCC for two further periods, the effect applying across tumour groups, though concentrated mostly on leukaemias and lymphomas. Sorahan et al.^41^ present the data for 1953–55 and review the literature, while Sorahan et al.^42^ analyse data for the years 1971–76 and discuss possible mechanisms for what may turn out to be a causal link.

#### Risks to sibs of children with cancer

In the first major paper from the OSCC referred to above, Stewart et al.^4^ summarised data on eight reports of possible deaths from malignant disease in sibs of the survey cases. In five of these they considered that the reports did indeed indicate that the sib died of malignant disease. In a subsequent progress report Barber and Spiers^31^ updated these results and reported 31 deaths from neoplasms compared with an expected number of 7.9, giving a RR of about four—though a later paper based on larger numbers and a more closely defined method of analysis gave different results.^43^ This latter paper, published at the time the Department of Social Medicine in Oxford was being transformed and the CCRG was opening, gave estimates of the risks to sibs of cases for various diagnostic groups. Excluding twins, cases of retinoblastoma (of which many are associated with RB1 gene germ cell mutations), and families with genetic disease having a recognised increased risk of childhood cancer, the calculated risk that a sib of a child with cancer would also be affected by cancer below age 15 years was double the normal risk. For genetic counselling, the estimates in this paper are to be preferred to earlier ones.

#### Childhood cancer in twins

Twins are less likely than singletons to develop childhood malignant disease. Hewitt et al.^44^ suggested that this was because a member of a pair affected in utero may have an increased risk of dying before the twin pregnancy is recognised as such. They argued that this conclusion was supported by the finding that the twin deficit applied especially to members of like-sex pairs, and that this could reflect prenatal selection against embryos with a disposition to develop cancer in childhood. Twin concordance, the likelihood of both members of a twin pair having childhood cancer, is discussed in Draper et al.;^1^ that discussion is based partly on findings from the OSCC.

#### Geographical studies

A number of geographical studies have been published using OSCC data; see, for example, Knox et al.^45^ on background radiation, Knox and Gilman^46^—one of a series of papers on clustering—and Knox,^47^ the last in a series of papers on environmental pollution. The geographical potential of the OSCC is limited, however, by having relatively imprecise address coding and incomplete case representation, particularly for the later years, when an increasing number of children have survived the disease. These studies may reasonably be regarded as less reliable than subsequent analyses of registration data as described in the companion paper.^1^

#### Collaborative study on radiation workers

In a collaborative study on the risk to the children of radiation workers,^48^ data from the OSCC were combined with data from the NRCT and from a separate Scottish study^49^ and used to assess the cancer risk to the children of exposed workers in radiation related industries. Records from the National Registry for Radiation Workers were used to identify the parents of cases and controls who were occupationally exposed prior to the conception of the child. The numbers of such parents linked were small and, as reported in Kendall et al.^2^ the results were not indicative of a risk: although there was an excess of radiation workers amongst the parents of cases, there was no indication of a dose–response effect. A follow-up paper by Sorahan et al.^50^ examined the timing of the workers’ exposure and found significant associations with exposure at conception and at diagnosis, but concluded that it was not possible to distinguish these effects.

## Discussion

### Current state of the data

The archiving project referred to above is still under way, though it is hoped to finish it during 2018–19. At this point, it is planned to lodge the available information in an electronic archive, possibly the Richard Doll Centenary Archive accommodated within the Nuffield Department of Population Health in Oxford. It is hoped that it would then be generally available, subject to the terms and conditions laid down by the UK data protection authorities. In addition to the computerised dataset and the digitised interview images, it is planned to include the hospital records referred to above. We believe that it would be scientifically beneficial if responsibility for the data could be assumed by an epidemiological unit with interests in paediatric oncology, so that licensed access to the available information could be maintained.

### Impact of the OSCC research

Without in any way wishing to diminish the impact of other surveys of childhood cancers, we believe that the OSCC, as the largest case-control survey of the diseases ever undertaken, has had a very significant impact on our understanding of their aetiology. The expectation that strong associations with exogenous factors, similar to those observed for many adult cancers, might exist has not been fulfilled and such associations as have been observed have been modest. This is true even for pre-natal X-raying—almost certainly the most important association reported by the OSCC.

This one finding, however, has had a very significant effect on our beliefs about the risk of low-dose radiation, particularly following more recent analyses endorsed by Richard Doll.^10^ In spite of initial resistance to acceptance of a causal relationship, the finding played a major part in the abandonment of routine ante-natal X-rays and their replacement by ultra-sound.^25^ Of possibly greater significance has been the impact on our understanding of the effects of low dose radiation and the widespread abandonment of threshold models of radiation carcinogenesis. Although practical considerations lead us to accept that some doses may safely be ignored—and indeed are unavoidable—it would now seem that no dose of ionising radiation entails zero risk. This observation may have little impact on a small scale of human exposure, but it acquires considerable significance when applied to the exposure of whole populations to small extra doses, as after a nuclear accident, for example.

Other associations ascertained from the OSCC have been less clear-cut, though there are certainly valuable pointers to the possible effects of some exogenous factors, including infectious organisms, certain classes of drugs taken in pregnancy and paternal smoking, as discussed above. The importance of genetic factors is clear, too, and estimates of familial risk are of considerable value for genetic counselling.

Accepting that the associations detected are fewer and weaker than would be expected for adult cancers is of value in itself, particularly as it has been possible to exclude a number of life-style and other factors that can worry mothers with affected children or with children as yet unborn. The foetus is well protected in pregnancy and it has become increasingly certain that few if any of the ordinary impacts of everyday life pose a risk of cancer in the unborn child.

It is clear that the total risk attributable to the associations identified remains very modest and the conclusion must be that the ‘cause’ of most cases is unknown, except to the extent that it would seem to be influenced by genetic attributes, endogenously determined, that are only slowly beginning to be understood. The value of the OSCC is clearly limited by the absence of genetic material; nevertheless the large number of possible associations and the descriptions of a significant number of cases, some of very rare tumours, suggest an enduring potential for continuing research.

## Electronic supplementary material

OSCC Publications
