# Supplementary material for: Childhood cancer research in Oxford I: the Oxford Survey of Childhood Cancers
Source: Br J Cancer. 2018 Aug 21;119(6):756–62. doi: 10.1038/s41416-018-0180-0 (PMC6173688; doi:10.1038/s41416-018-0180-0)
Supplement: Supplementary file 1 — OSCC Publications [file 41416_2018_180_MOESM1_ESM.rtf]

List of publications resulting from the Oxford Survey of 
Childhood Cancers

These publications are those deemed to have reported actual data from the survey or to have made a contribution to evaluating the impact of the data.  A few minor publications that are now difficult to locate have been omitted.  The papers are ordered by year of publication and by authors' names within year.


1956-64

Stewart A, Webb J, Giles D, Hewitt D (1956) Malignant disease in childhood and diagnostic irradiation in utero. Lancet 271(6940): 447

Stewart A (1957) A current survey of malignant disease in children. Proc R Soc Med 50(4): 251-2

Stewart A, Webb J, Hewitt D (1958) A survey of childhood malignancies. Br Med J 1(5086): 1495-508

Stewart A (1961) Aetiology of Childhood Malignancies. Br Med J 1(5224): 452-60

Stewart A (1961) Aetiology of Leukaemia. Br Med J 1(5224): 507

Wise ME (1961) Irradiation and leukaemia. Br Med J 2(524): 48-49

Stewart A, Barber R (1962) Survey of childhood malignancies. Public Health Rep 77: 129-39

Stewart A, Hewitt D (1963) Oxford Survey of Childhood Cancers: progress report. I. Mon Bull Minist Health Public Health Lab Serv 22: 182-92

Barber R, Spiers P (1964) Oxford Survey of Childhood Cancers: progress report. II. Mon Bull Minist Health Public Health Lab Serv 23: 46-52

Hewitt D, Spiers PS (1964) Low prevalence of A-antigen in children with acute myeloid leukaemia. Lancet 284(7350): 93-94

1965-68

Lashof JC, Stewart A (1965) Oxford Survey of Childhood Cancers: progress report. 3. Leukemia and Down's syndrome. Mon Bull Minist Health Public Health Lab Serv 24: 136-43

Stewart A, Hewitt D (1965) Leukaemia Incidene in Children in Relation to Radiation Exposure in Early Life. In Current Topics in Radiation Research, Ebert M, Howard A (eds) Vol. I, Chapter VI, pp 221-253. Amsterdam: North Holland

Stewart AM, Hewitt D (1965) Aetiology of childhood leukaemia. Lancet 2(7416): 789-90

Hewitt D (1966) Epidemiologic detection of agents whose effects depend on host susceptibility. J Chronic Dis 19(10): 1059-66

Hewitt D, Lashof JC, Stewart AM (1966) Childhood cancer in twins. Cancer 19(2): 157-61

Hewitt D, Sanders B, Stewart A (1966) Oxford Survey of Childhood Cancers: progress report. IV. Reliability of data reported by case and control mothers. Mon Bull Minist Health Public Health Lab Serv 25: 80-5

Stewart A (1967) Epidemiology of Childhood Cancers. In The prevention of cancer, Raven RW, Roe FJC (eds), Chapter 56, pp 352-358. London Butterworths 

Stewart A, Kneale GW (1968) Cancer risk in obstetric radiography. Lancet 1(7537): 305

Stewart A, Kneale GW (1968) Changes in the cancer risk associated with obstetric radiography. Lancet 1(7534): 104-7

Stewart AM (1968) Leukemia and other neoplasms in childhood following radiation exposure in utero--a general survey of present knowledge. Br J Radiol 41(489): 718-9

Stewart AM, Draper GJ (1968) X-rays and childhood cancer. Lancet 2(7572): 828-9

Stewart AM, Ledlie EM (1968) Cancer in children. Lancet 2(7565): 453-4

1969-70

Stewart A, Kneale GW (1969) Role of local infections in the recognition of haemopoietic neoplasms. Nature 223(5207): 741-2

Stewart AM (1969) Measuring latent periods of juvenile cancers. Proc R Soc Med 62(7): 684

Stewart AM (1969) Radiogenic Cancers of Childhood. In Radiation Biology of the Fetal and Juvenile Mammal, Sikov MR, Mahlum DD (eds) pp 681-692. Proceedings of the Ninth Annual Hanford Biology Symposium, Richland, Washington: US Atomic Energy Commission

Hewitt D, Stewart A (1970) Relevance of twin data to intrauterine selection: special case of childhood cancer. Acta geneticae medicae et gemellologiae 19(1): 83-6

Ledlie EM, Mynors LS, Draper GJ, Gorbach PD (1970) Natural history and treatment of Wilms' tumour:  an analysis of 335 cases occurring in England and Wales 1962-6. Br Med J 4(5729): 195-200

Stewart A, Kneale GW (1970) Prenatal radiation exposure and childhood cancer. Lancet 1(7684): 1189-90

Stewart A, Kneale GW (1970) Radiation dose effects in relation to obstetric x-rays and childhood cancers. Lancet 1(7658): 1185-8

Stewart A, Kneale GW (1970) The role of local infections in the recognition of haemopoietic neoplasms. Br J Prev Soc Med 24(1): 65

Stewart AM, Draper GJ (1970) B.C.G. vaccination and leukaemia mortality. Lancet 2(7680): 983-4

Stewart AM, Kneale GW (1970) Age-distribution of cancers caused by obstetric x-rays and their relevance to cancer latent periods. Lancet 2(7662): 4-8

1971-72

Kneale GW (1971) Excess sensitivity of pre-leukaemics to pneumonia.  A model situation for studying the interaction of infectious disease with cancer. Br J Prev Soc Med 25(3): 152-159

Kneale GW (1971) Problems arising in estimating from retrospective survey data the latent periods of juvenile cancers initiated by obstetric radiography. Biometrics 27(3): 563-90

Stewart A (1971) Low dose radiation cancers in man. Adv Cancer Res 14: 359-90

Stewart A (1971) Oxford survey of childhood cancers. Nurs Mirror Midwives J 133(17): 25-6

Stewart A (1971) Prenatal radiation exposure and childhood cancer. Lancet 1(7702): 751-2

Stewart A, Barber R (1971) The epidemiological importance of childhood cancers. Br Med Bull 27(1): 64-70

Stewart A, Kneale GW (1971) Prenatal radiation exposure and childhood cancer. Lancet 1(7688): 42-3

Stewart AM, Draper GJ, Kneale GW (1971) Childhood cancer following obstetric radiography. Lancet 2(7739): 1424

Draper GJ, Gorbach PD (1972) Epidemiological study of the progression of cancer, illustrated by Wilms's tumour. Proc R Soc Med 65(3): 242-4

Gehan EA, Kneale GW (1972) Relationship of Prenatal Irradiation to Death from a Malignant Disease. Biometrics 28(1): 239-245

Stewart A (1972) Myeloid leukaemia and cot deaths. Br Med J 4(5837): 423

Stewart A (1972) Prenatal x-ray exposure and childhood neoplasms. Br J Radiol 45(538): 794

Stewart A (1972) Tissue ageing as a factor in juvenile cancers. Proc R Soc Med 65(3): 245-6

1973-74

Bithell JF, Draper GJ, Gorbach PD (1973) Association between malignant disease in children and maternal virus infections. Br Med J 1(5855): 706-708

Dixon WJ, Landau E, Murthy VK, Stewart A (1973) Discussion of leukemia irradiation studies. J Natl Cancer Inst 50(4): 1085-6

Draper GJ (1973) Cancer in Children. In Cancer at the crossroads and the Challenge for the Future, Raven RW (ed), pp 31-37. London: Heinemann

Draper GJ (1973) Down's syndrome and acute leukaemia Lancet 1(7793): 53-53

Stewart A (1973) The carcinogenic effects of low level radiation. A re-appraisal of epidemiologists methods and observations. Health Phys 24(2): 223-40

Stewart A (1973) Twins as markers of the value of obstetric radiography (abstract). Br J Prev Soc Med 27(1): 69

Stewart A, Kneale GW (1973) Childhood cancer following obstetric radiography. Health Phys 24(3): 359

Stewart AM (1973) Cancer as a cause of abortions and stillbirths: the effect of these early deaths on the recognition of radiogenic leukaemias. Br J Cancer 27(6): 465-72

Stewart AM (1973) An epidemiologist takes a look at radiation risks. In Reports of US Dept of Hlth Ed and Welfare Vol. FDA 73-8024. Rockville, Md.: Bureau of Radiological Health

Stewart AM, Lennox EL, Sanders BM (1973) Group characteristics of children with cerebral and spinal cord tumours. Br J Cancer 28(6): 568-574

Hinds SW, Kinnier Wilson LM, Draper GJ (1974) Childhood malignant disease in Britain 1953-1971. Health Soc Serv J(April 20): 880-881

Kinnier Wilson LM, Draper GJ (1974) Neuroblastoma, its natural history and prognosis: a study of 487 cases. Br Med J 3(5926): 301-307

Leighton P, Smith PG, Draper GJ, Pike MC (1974) Malignant disease in the parents of children dying of Hodgkin's disease. Br J Cancer 30(4): 373-375

Mole RH (1974) Antenatal irradiation and childhood cancer: Causation or coincidence? Br J Cancer 30(3): 199-208

Stewart A (1974) Letter: Factors affecting recognition of infant leukaemias and lymphomas. Br Med J 2(5919): 611-2

1975-77

Bithell JF, Stewart AM (1975) Pre-natal irradiation and childhood malignancy: a review of British data from the Oxford Survey. Br J Cancer 31(3): 271-87

Draper GJ (1975) Epidemiological evidence concerning the time of initiation of cancer in children. In XI International Cancer Congress: Series No. 350, Bucalossi  P, Veronesi U, Cascinell N (eds) Vol. 2, Chemical and viral oncogenesis. pp 150-155. Florence: Amsterdam: Excerpta Medica 

Kinnier Wilson LM (1975) Some aspects of epidemiology in paediatric oncology. Proc Roy Soc Med 68: 657-659


Lennox EL, Draper GJ, Sanders BM (1975) Retinoblastoma: a study of natural history and prognosis of 268 cases. Br Med J 3(5986): 731-734

Stewart A (1975) Infant leukaemias and cot deaths. Br Med J 2(5971): 605-7

Kneale GW, Stewart AM (1976) Letter: Antenatal radiography and the ten-day rule. Lancet 1(7967): 1021

Kneale GW, Stewart AM (1976) Mantel-Haenszel analysis of Oxford data. I. Independent effects of several birth factors including fetal irradiation. J Natl Cancer Inst 56(5): 879-83

Kneale GW, Stewart AM (1976) Mantel-Haenszel analysis of Oxford data. II. Independent effects of fetal irradiation subfactors. J Natl Cancer Inst 57(5): 1009-14

Draper GJ, Heaf MM, Kinnier Wilson LM (1977) Occurrence of childhood cancers among sibs and estimation of familial risks (abstract). J Med Genet 14: 81-90

Kneale GW, Stewart AM (1977) Age variation in the cancer risks from foetal irradiation. Br J Cancer 36(4): 501-10

Stewart A (1977) Factors affecting the recognition of childhood cancers: Respiratory infections, cot deaths, and season of birth. Pediatrics Digest 19(January): 9-20

1978-80

Kneale G, Stewart AM (1978) Low-dose radiation. Lancet 2(8083): 262-3

Kneale GW, Stewart AM (1978) Pre-cancers and liability to other diseases. Br J Cancer 37(3): 448-57

Lennox EL, Stiller CA, Morris Jones PH, Kinnier Wilson LM (1979) Nephroblastoma: treatment during 1970-3 and the effect on survival of inclusion in the first MRC trial. Br Med J 2(6190): 567-569

Sanders BM, Draper GJ (1979) Childhood cancer and drugs in pregnancy. Br Med J 1(6165): 717-718

Blot WJ, Draper GJ, Kinlen L, Kinnier Wilson LM (1980) Childhood cancer in relation to prenatal exposure to chickenpox. Br J Cancer 42: 342-344

Blot WJ, Stiller CA, Kinnier Wilson LM (1980) Oral clefts and childhood cancer. Lancet 315: 722

Kneale GW, Stewart AM (1980) Pre-conception X-rays and childhood cancers. Br J Cancer 41(2): 222-6

Knox EG, Stewart A, Kneale G (1980) Childhood leukaemia and mother-foetus infection. Br J Cancer 42(1): 158-161

Marsden HB, Lennox EL, Lawler W, Kinnier-Wilson LM (1980) Bone metastases in childhood renal tumours. Br J Cancer 41(6): 875-879

Stewart A (1980) Childhood cancers and the immune system. Cancer Immunol Immunother 9(1-2): 11-14

Stiller CA, Lennox EL (1980) Nephroblastoma in infants, 1969-75: variations in treatment and survival. Br Med J 281(6250): 1246-1248

1981-84

Kinnier-Wilson LM, Kneale GW, Stewart AM (1981) Childhood cancer and pregnancy drugs. Lancet 2(8241): 314-5

Kinnier-Wilson LM, Kneale GW, Stewart AM (1981) Childhood cancer and pregnancy drugs (corr.). Lancet 2(8241): 314-315

Kneale GW, Mancuso TF, Stewart AM (1981) Hanford radiation study III: a cohort study of the cancer risks from radiation to workers at Hanford (1944-77 deaths) by the method of regression models in life-tables. Br J Ind Med 38(2): 156-66

Stiller CA, Kinnier-Wilson LM (1981) Down syndrome and leukaemia. Lancet 318(8259): 1343

Kneale GW, Stewart AM (1982) Do childhood cancers result from pre-natal x-rays? Health Phys 42(3): 388-90

Stewart AM, Kneale GW (1982) The immune system and cancers of foetal origin. Cancer Immunol Immunother 14(2): 110-116

Swerdlow AJ, Stiller CA, Kinnier Wilson LM (1982) Prenatal factors in the aetiology of testicular cancer: an epidemiological study of childhood testicular cancer deaths in Great Britain, 1953-73. J Epidemiol Community Health 36(2): 96-101

Knox EG, Stewart AM, Kneale GW (1983) Foetal infection, childhood leukaemia and cancer. Br J Cancer 48(6): 849-852

Stiller CA, Lennox EL (1983) Childhood medulloblastoma in Britain 1971-77: analysis of treatment and survival. Br J Cancer 48(6): 835-841

Kinnier Wilson LM, Waterhouse JAH (1984) Obstetric ultrasound and childhood malignancies. Lancet 324(8410): 997-999

Knox EG, Marshall T, Barling R (1984) Leukaemia and childhood cancer in twins. J Epidemiol Community Health 38(1): 12-16

1985-89

Ilgren EB, Kinnier Wilson LM, Stiller CA (1985) Gliomas in neurofibromatosis: a series of 89 cases with evidence for enhanced malignancy in associated cerebellar astrocytomas. Pathol Annu 20(Pt 1): 331-358

Kneale GW, Stewart AM (1986) Prenatal x rays and cancers: further tests of data from the Oxford Survey of Childhood Cancers. Health Phys 51(3): 369-76


Kneale GW, Stewart AM, Kinnier Wilson LM (1986) Immunizations against infectious diseases and childhood cancers (abstract). Cancer Immunol Immunother 21(2): 129-132

Eisenberg DE, Sorahan T (1987) Birth weight and childhood cancer deaths. J Natl Cancer Inst 78(6): 1095-1100

Knox EG, Stewart AM, Kneale GW, Gilman EA (1987) Prenatal irradiation and childhood cancer. J Radiol Prot 7(4): 177-89

Stiller CA, Lennox EL, Kinnier Wilson LM (1987) Incidence of cardiac septal defects in children with Wilms' tumour and other malignant diseases. Carcinogenesis 8(1): 129-132

Bithell JF, Stiller CA (1988) A new calculation of the carcinogenic risk of obstetric X-raying. Stat Med 7(8): 857-864

Gilman EA, Kneale GW, Knox EG, Stewart AM (1988) Pregnancy x-rays and childhood cancers: effects of exposure age and radiation dose. J Radiol Prot 8(1): 3-8

Knox EG, Stewart AM, Gilman EA, Kneale GW (1988) Background radiation and childhood cancers. J Radiol Prot 8(1): 9-18

Bithell JF (1989) Epidemiological studies of children irradiated in utero. In Low dose radiation: biological bases of risk assessment, Baverstock K, Stather J (eds) pp 77–87. L.H. Gray conference, Oxford (UK). London: Taylor & Francis

Bithell JF (1989) Statistical issues in assessing the evidence associating obstetric irradiation and childhood malignancy. In Neue Bewertung des Strahlenrisikos: Niedrigdosis-Strahlung und Gesundheit, Lengfelder E, Wendhausen H (eds) pp 53–60. Internationale Konferenz der Gesellschaft für Strahlenschutz e. V. Kiel, 1992. Munich: MMV Medizin

Gilman EA, Kinnier Wilson LM, Kneale GW, Waterhouse JA (1989) Childhood cancers and their association with pregnancy drugs and illnesses. Paediatr Perinat Epidemiol 3(1): 66-94

Gilman EA, Stewart AM, Knox EG, Kneale GW (1989) Trends in obstetric radiography, 1939-81. J Radiol Prot 9(2): 93

Muirhead CR, Kneale GW (1989) Prenatal irradiation and childhood cancer. J Radiol Prot 9(3): 209-212

1992-97

Draper GJ, Kendall GM, Muirhead CR, Sorahan T, Fox AJ, Kinlen LJ (1992) Cancer in the children of radiation workers. Radiol Prot Bull 129: 10-14

Sorahan T, Stewart AM (1993) Retinoblastoma and fetal irradiation. Br Med J 307(6908): 870

Gilman EA, Knox EG (1995) Childhood cancers: space-time distribution in Britain. J Epidemiol Community Health 49(2): 158-163

Sorahan T, Lancashire R, Prior P, Peck I, Stewart A (1995) Childhood cancer and parental use of alcohol and tobacco. Ann Epidemiol 5(5): 354-359

Sorahan T, Lancashire R, Stewart A, Peck I (1995) Pregnancy ultrasound and childhood cancer: a second report from the Oxford Survey of Childhood Cancers. Br J Obstet Gynaecol 102(10): 831-832

Stewart A (1995) Alternative sources of risk estimates for cancer effects of radiation. Mt Sinai J Med 62(5): 380-5

Stewart A (1995) Childhood cancers and competing causes of death. Leuk Res 19(2): 103-11

Draper GJ, Sanders BM, Lennox EL, Brownbill PA (1996) Patterns of childhood cancer among siblings. Br J Cancer 74(1): 152-158

Knox EG, Gilman EA (1996) Spatial clustering of childhood cancers in Great Britain. J Epidemiol Community Health 50(3): 313-319

Draper GJ, Little MP, Sorahan T, Kinlen LJ, Bunch KJ, Conquest AJ, Kendall GM, Kneale GW, Lancashire RJ, Muirhead CR, O'Connor CM, Vincent TJ (1997) Cancer in the offspring of radiation workers: a record linkage study. Br Med J 315(7117): 1181-1188

Draper GJ, Little MP, Sorahan T, Kinlen LJ, Bunch KJ, Conquest AJ, Kendall GM, Kneale GW, Lancashire RJ, Muirhead CR, O'Connor CM, Vincent TJ, Thomas JM, Goodill AA, Vokes J, Haylock RGE (1997) NRPB-R298: Cancer in the Offspring of Radiation Workers - a Record Linkage Study, NRPB-R298 edn. Didcot: NRPB

Kneale G (1997) Risk of childhood cancer from fetal irradiation: 2. Br J Radiol 70(835): 770-71

Knox EG, Gilman EA (1997) Hazard proximities of childhood cancers in Great Britain from 1953-80. J Epidemiol Community Health 51(2): 151-159

Sorahan T, Lancashire RJ, Hultén MA, Peck I, Stewart AM (1997) Childhood cancer and parental use of tobacco: deaths from 1953 to 1955. Br J Cancer 75(1): 134-138

Sorahan T, Prior P, Lancashire RJ, Faux SP, Hultén MA, Peck IM, Stewart AM (1997) Childhood cancer and parental use of tobacco: deaths from 1971 to 1976. Br J Cancer 76(11): 1525-1531

Stewart AM (1997) Risk of childhood cancer from fetal irradiation: 1. Br J Radiol 70(835): 769-70; author reply 771

Wakeford R, Doll R, Bithell JF (1997) Childhood cancer and intrauterine irradiation. In Health effects of low dose radiation : challenges of the 21st century : proceedings of the conference organized by the British Nuclear Energy Society. Stratford-upon-Avon, UK: Thomas Telford

Wakeford R, Doll R, Bithell JF (1997) The risk of childhood cancer from low doses of ionising radiation received in utero. In Low doses of ionoizing radiation: biological effects and regulatory control Vol. IAEA-TECDOC-976. pp 392-394. Vienna: International Atomic Energy Agency


1998-2018

Gilman EA, Knox EG (1998) Geographical distribution of birth places of children with cancer in the UK. Br J Cancer 77(5): 842-849

Gilman EA, Sorahan T, Lancashire RJ, Lawrence GM, Cheng KK (1998) Seasonality in the presentation of acute lymphoid leukaemia (corres.). Br J Cancer 77(4): 677-678

Kneale GW (1998) Risk of childhood cancer from fetal irradiation. Br J Radiol 71(844): 460-461

Knox EG, Gilman EA (1998) Migration patterns of children with cancer in Britain. J Epidemiol Community Health 52(11): 716-726

Sorahan T, Hamilton L, Gardiner K, Hodgson JT, Harrington JM (1999) Maternal occupational exposure to electromagnetic fields before, during, and after pregnancy in relation to risks of childhood cancers: findings from the Oxford survey of childhood cancers, 1953-1981 deaths. Am J Ind Med 35(4): 348-357

Knox EG (2000) Childhood cancers, birthplaces, incinerators and landfill sites. Int J Epidemiol 29(3): 391-397

Lancashire RJ, Sorahan T (2003) Breastfeeding and childhood cancer risks: OSCC data. Br J Cancer 88(7): 1035-1037

Sorahan T, Haylock RGE, Muirhead CR, Bunch KJ, Kinlen LJ, Little MP, Draper GJ, Kendall GM, Lancashire RJ, English MA (2003) Cancer in the offspring of radiation workers: an investigation of employment timing and a reanalysis using updated dose information. Br J Cancer 89(7): 1215-1220

Wakeford R, Little MP (2003) Risk coefficients for childhood cancer after intrauterine irradiation: a review. Int J Radiat Biology 79(5): 293-309

Sorahan T, Lancashire RJ (2004) Parental cigarette smoking and childhood risks of hepatoblastoma: OSCC data. Br J Cancer 90(5): 1016-1018

Knox EG (2005) Childhood cancers and atmospheric carcinogens. J Epidemiol Community Health 59(2): 101-105

Knox EG (2005) Oil combustion and childhood cancers. J Epidemiol Community Health 59(9): 755-760

Knox EG (2006) Roads, railways, and childhood cancers. J Epidemiol Community Health 60(2): 136-141

Bithell JF, Draper GJ, Sorahan T, Stiller CA (2018) Childhood Cancer in Oxford I: The Oxford Survey of Childhood Cancers. Submitted
